# Oral health, dysbiotic oral microbiome, and hypertension in Sub-Saharan Africa: a scoping review of current evidence

**DOI:** 10.1097/MS9.0000000000005750

**Published:** 2026-10-28

**Authors:** Agnes Gatarayiha, Pascal Ubuzima, Eugene Nshimiyimana, Donat Uwayezu, Léon Mutesa

**Affiliations:** a Preventive and Community Dentistry, School of Dentistry, College of Medicine and Health Sciences, University of Rwanda, Kigali, Rwanda; b Department of Orthodontics, Affiliated Hospital of Stomatology, Anhui Medical University, Hefei, Hefei, Anhui, China; c Center for Human Genetics, College of Medicine and Health Sciences, University of Rwanda, Kigali, Rwanda

**Keywords:** dysbiotic oral microbiome, hypertension, oral health, periodontal diseases, Sub-Saharan Africa

## Abstract

**Background:**

Hypertension (HTN) is a significant public health concern globally, with Sub-Saharan Africa (SSA) bearing a considerable burden. Dysbiosis of the oral microbiome may play a role in the pathogenesis of HTN, with a scarcity of evidence in SSA. The aim of this review was to explore the existing literature on the influence of oral health and dysbiotic oral microbiomes on the development of HTN in SSA countries.

**Methods:**

A comprehensive search across electronic databases, including PubMed, Google Scholar, Scopus, ScienceDirect, and Web of Science, was conducted from 2000 up to December 2025. Studies involving SSA populations and addressing the role of the oral microbiome in HTN were included.

**Results:**

The review identified 12 studies investigating oral microbiota dysbiosis and its association with HTN in six SSA countries (South Africa, Nigeria, Tanzania, Senegal, Ghana, and Cameroon). Of these, 10 studies assessed periodontal diseases using clinical indices as a proxy for dysbiosis, 1 study assessed dental caries, while only a single study performed direct microbial analysis (using 16S rRNA gene sequencing). Eight studies reported quantitative effect estimates: odds ratios for the association between periodontal disease or gingivitis and HTN ranged from 1.5 to 4.30 (all *P* < 0.05), while one study reported an association between dental caries and HTN (odds ratio, 0.72–0.73). The reported prevalence of HTN ranged between 19.7 and 91.8%.

**Conclusion:**

This scoping review finds that most available evidence from SSA supports an association between periodontal disease and HTN. However, direct evidence regarding the oral microbiome itself remains very limited, with only one study performing species-level microbial analysis.

## Introduction

Hypertension (HTN) is characterized by a diastolic blood pressure (DBP) of at least 90 mmHg and/or a systolic blood pressure (SBP) of at least 140 mmHg, or by the consistent use of antihypertensive drugs^[^1^]^. It is a significant risk factor for cardiovascular diseases (CVD), stroke, and other health complications such as chronic kidney disease, atrial fibrillation, heart failure, and other potentially fatal illnesses^[^2–4^]^. The high prevalence of HTN and CVD has been reported across Sub-Saharan Africa (SSA)^[^5,6^]^. In Africa, the prevalence of HTN is expected to reach 216.8 million cases by 2030^[^7^]^. This expanding HTN epidemic in SSA is characterized by high prevalence rates and restricted access to quality healthcare resources^[^8^]^.

HIGHLIGHTS

- This review synthesizes evidence from Sub-Saharan Africa linking oral microbiome dysbiosis to hypertension.
- Across 12 included studies, hypertension prevalence in the studied populations ranged from 19.7 to 91.8%.
- Dysbiosis, marked by altered microbial diversity and composition, is associated with hypertension in this region.
- Proposed mechanistic pathways connect oral dysbiosis to systemic inflammation and endothelial dysfunction.
- The findings highlight oral health as a potential modifiable target for hypertension prevention and management in Sub-Saharan Africa.

In 2005, CVDs were estimated to have caused 17.5 million deaths, and by 2021 this number had risen to 20.5 million CVD-related deaths, with over 80% occurring in middle- and low-income countries^[^5^]^. In 2019, 18.6 million deaths were due to CVD worldwide^[^9^]^. In 2015, SBP >115 mmHg was estimated to have caused 8.5 million deaths, of which low- and middle-income countries (LMICs) accounted for 88%^[^10^]^. In 2010, an increase to 67% since 1990 was reported, with HTN as the leading risk factor^[^11^]^. Tanzania, a country in SSA, reported that 15% of overall deaths and 34% of deaths from non-communicable diseases (NCDs) were attributable to HTN-associated illnesses, including stroke^[^12^]^.

The key characteristics linked to the prevalence of HTN and its distribution differences across and between SSA nations include use of tobacco products, excessive alcohol use, inactivity, poor nutrition (high salt and low fruit and vegetable intake), obesity, aging, increased adiposity and underweight, and educational and/or financial status^[^13–15^]^. Older age and primary education were found to be associated with higher and lower HTN, respectively, in East and West Africa^[^16^]^. This situation negatively affects the prevention, detection, management, and control of HTN, calling for immediate attention. A scoping review conducted on job stress and HTN in 2023 reported that among SSA workers aged 18–79, between 14.3 and 45.9% have HTN, and a sizable portion of them (35.4–70.6%) are not aware that they have the condition^[^17^]^.

The oral microbiome refers to the diverse community of microorganisms, including bacteria, viruses, fungi, archaea, and protozoa^[^18^]^. These microbes can colonize a variety of oral surfaces, including the palate, teeth, dental prostheses, tonsils, tongue, and buccal mucosa. Stable diversity and composition indicate that these species are in balance within a healthy ecosystem^[^19^]^. However, a disruption of this balance can result in dysbiosis. Dysbiosis alters the overall composition of the oral microbiome by reducing beneficial bacteria and increasing commensal microorganisms^[^20^]^. This imbalance can result from various factors, including dietary habits, inflammatory processes, altered salivary flow, systemic health conditions, lifestyle choices such as alcohol consumption, smoking, antibiotic use, infections, and stress^[^21^]^.

The oral microbiome performs essential homeostatic roles in the body. For example, because of its ability to reduce dietary nitrate, the oral microbiome can increase systemic nitrite levels. This, in turn, raises nitric oxide (NO) concentrations. NO preserves vascular homeostasis due to its vasodilatory and anti-inflammatory functions^[^22,23^]^. Furthermore, dysbiosis of the subgingival microbiome triggers the innate immune response, which affects the vascular endothelium. Periodontitis and dental caries are the two most common oral diseases worldwide, affecting people globally and are more prevalent on the African continent^[^24,25^]^. Although the bulk of prior research has focused on the role of bacteria, recent investigations have also demonstrated the involvement of the oral microbiome in the development of HTN^[^26^]^.

Dysbiotic oral microbiota may release oral microbiome products and inflammatory molecules into the bloodstream and digestive tract. This affects blood vessel function and contributes to HTN, where altered microbial communities could also impact systemic inflammation and endothelial dysfunction^[^27,28^]^. HTN imposes a high burden in SSA, with poor detection, treatment, and control rates. It was reported that 66% of hypertensive individuals were unaware of their condition, 82% were not treated, and 93% were not controlled^[^11^]^. Therefore, a research gap exists, especially in SSA, regarding microbiome studies related to diseases of public health importance^[^29^]^.

Additionally, as far as we are aware, there are few studies examining how dysbiosis of the oral microbiome contributes to HTN in SSA nations. This is important for understanding local health disparities as well as developing targeted interventions. Therefore, the purpose of this scoping review was to explore existing literature on the influence of oral health and dysbiotic oral microbiomes on the development of HTN in SSA countries. Future studies should focus on developing collaborations, building capacity, and supporting microbiome research across Africa.

## Methods

### Research and protocol registration

This scoping review adopted the design proposed by Arksey and O’Malley^[^30^]^ and was reported in accordance with the Preferred Reporting Items for Systematic Reviews and Meta-Analyses extension for Scoping Reviews (PRISMA-ScR) checklist^[^31^]^.

### Research question

What is the current evidence on the association between the oral microbiome and the development of HTN in SSA, and how do microbial alterations in individuals with both conditions compare with those in other African regions and global populations?

### Search strategies

A comprehensive search was conducted across electronic databases, including PubMed, Google Scholar, Scopus, ScienceDirect, and Web of Science. Relevant published articles in English from 2000 to December 2025 were included in this review.

The search terms used to retrieve the relevant articles are described in Supplemental Digital Content Table S1, available at: https://links.lww.com/MS9/B424. MeSH terms used are as follows: “Dysbiosis,” “Oral microbiome,” “Periodontal diseases,” “Dental caries,” “Tooth decay,” “HTN,” “High blood pressure,” “CVD,” and “SSA.” Boolean operators such as “AND” and “OR” were applied to identify related articles. Additionally, reference lists of the relevant retrieved articles were reviewed. Moreover, the search was guided by PICO (Population, Intervention, Comparison, and Outcomes) for the eligibility criteria.

**Population**: Subjects in SSA who have been diagnosed with oral conditions linked to a dysbiotic oral microbiome, such as periodontal disease or other oral inflammatory conditions.

**Intervention/exposure**: Subjects who have developed HTN, defined as having a DBP of ≥90 mmHg and/or an SBP of ≥140 mmHg, or who are consistently using antihypertensive medication in SSA.

**Comparison**: Subjects in SSA were categorized as normotensive, defined as having normal blood pressure (SBP < 140 mmHg and DBP < 90 mmHg) and no history of HTN or use of antihypertensive medication.

**Outcome**: The relationship between a dysbiotic oral microbiome and the development of HTN in SSA is evaluated, based on evidence that oral conditions such as periodontal diseases have been linked to CVDs, and on the association of poor systolic and DBP with dental caries in patients.

#### Inclusion criteria

The included studies encompass research articles focusing on oral microbiome dysbiosis characteristics, periodontitis and other periodontal diseases, and HTN, conducted in SSA countries, involving participants of any age, and employing study designs such as cross-sectional studies, cohort studies, case-control studies, and clinical trials.

#### Exclusion criteria

The excluded studies included those involving nonhuman subjects, animal studies, and laboratory studies. Articles for which we could not retrieve the full text, reviews, case series, case reports, short communications, letters to the editor, and conference abstracts with no original data were also excluded.

### Screening and data extraction

Two independent reviewers (P.U. and E.N.) screened the titles to remove duplicates and screened the abstracts against the inclusion criteria. An independent full-text assessment was done following the inclusion and exclusion criteria. Where conflicts arose, discussions were held, and complete consensus was reached. The reviewers (A.G., P.U., and E.N.) independently performed the data extraction to gather data from the 12 included studies. This included the authors' names, publication year, study population, objective, study design, methods used to collect relevant data, findings, and conclusions.

## Results

### Selection of the studies

During the literature search, 28 333 records were identified. After removing 25 883 duplicates and excluding records for other reasons, the remaining 2450 articles underwent screening based on their titles and abstracts, and 2200 records were excluded. Subsequently, 67 full-text articles were assessed for eligibility. Of these, 55 articles were excluded as they did not meet the predetermined inclusion criteria. Ultimately, 12 articles were included in this review, as shown in the PRISMA flowchart (Fig. 1).

**Figure 1.**
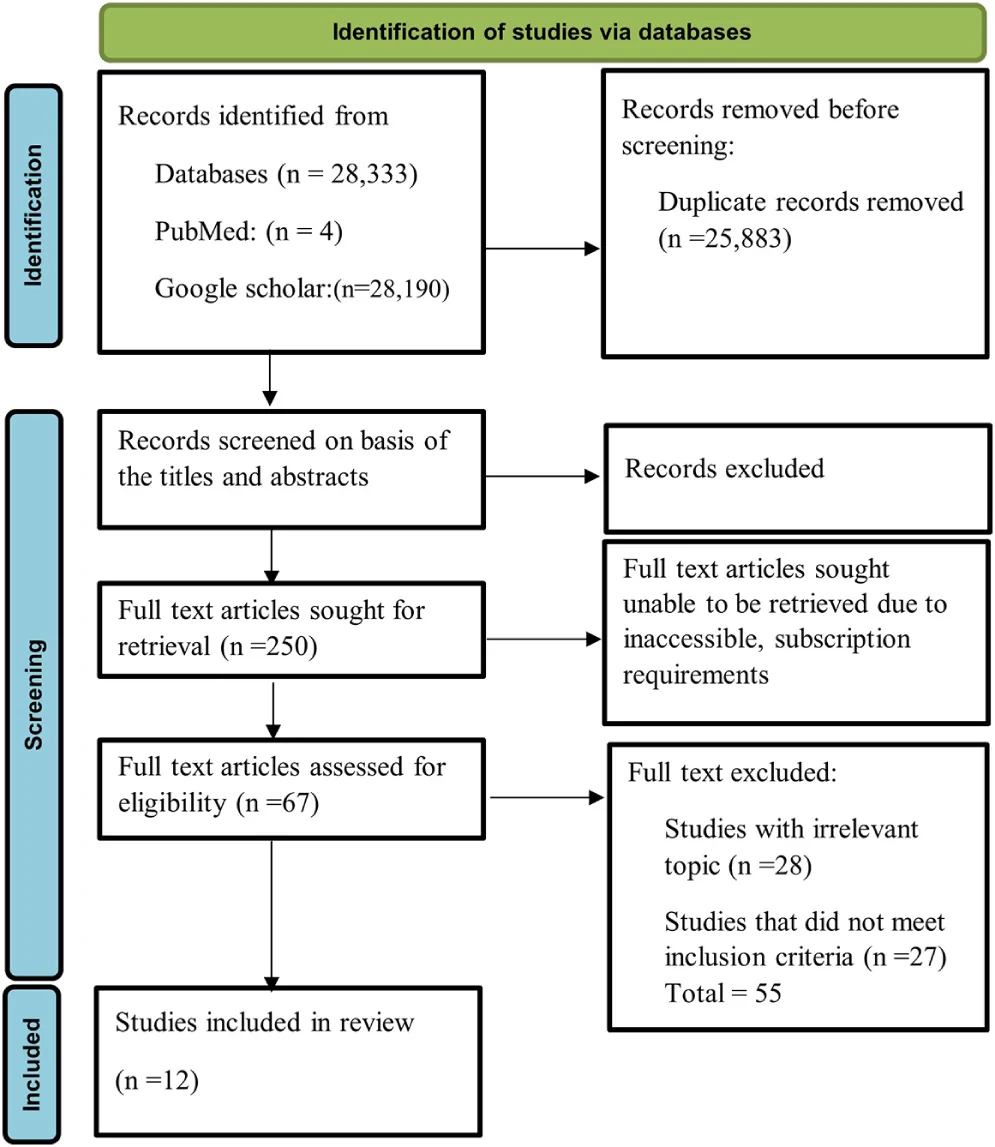
PRISMA flowchart.

### Characteristics of the included studies

In this review, the dysbiotic oral microbiome and HTN in SSA have been examined in 12 studies. These studies have a total sample size of 5130 subjects aged 18 years and above, with individual study sample sizes ranging from 80 to 1910 individuals. Three of the included studies are case-control studies^[^32–34^]^, 8 studies are cross-sectional^[^35–42^]^, and one is a prospective observational study^[^43^]^. An overview of the included studies that showed the association between oral microbiome dysbiosis and HTN in SSA is shown in Table 1. The included studies and their corresponding SSA countries are presented in Figure 2.

**Figure 2.**
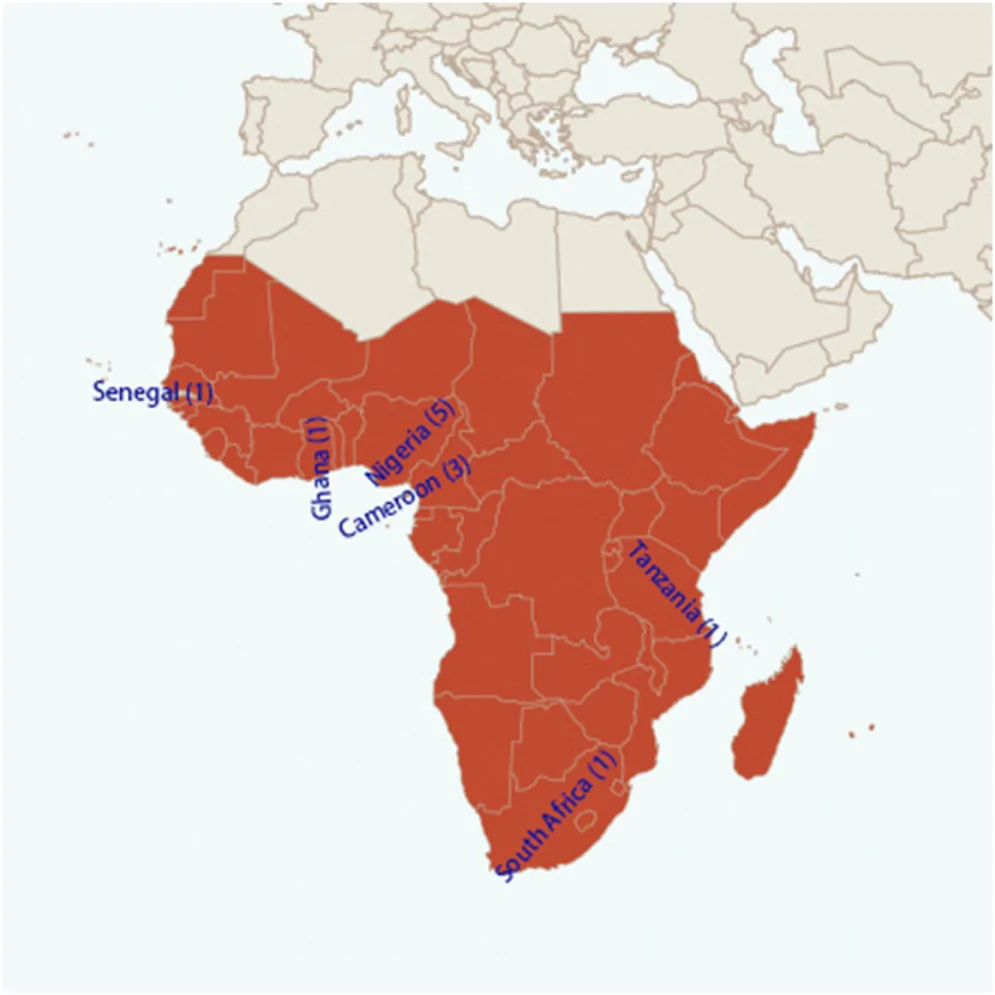
Sub-Saharan countries with the number of studies included in this review (in parentheses).

**Table 1:** The overview of included studies that showed the association between oral microbiome dysbiosis and hypertension in SSA countries.

Author/year	Country	Sample size	Ages of participants	Study design	Methods	Reported prevalence of hypertension or species identified	Conclusion
Leye *et al* ^[^32^]^	Senegal	246	58 ± 11.4 years (cases) and 34.5 ± 14.2 years (controls)	Case-control study	Sociodemographic characteristics Lifestyle and risk factors Data on hypertension and periodontal disease, including: Plaque index Papillary bleeding index Clinical attachment loss Pocket depth Community periodontal index and treatment needs (CPITN)	246 patients (123 hypertensive patients and 123 non-hypertensive patients)	Periodontal status significantly influences the occurrence of hypertension. An integrated approach, considering oral-dental factors, is essential in the treatment of hypertension.
Oyapero *et al* ^[^33^]^	Nigeria	268	18 years and older	Cross-sectional study	Structured interviewer-administered questionnaires Cardiovascular disease risk factors and specific diagnosis Anthropometric measurements Periodontal and caries status	91.8%	Cardiovascular disease (CVD) risk factors – including increasing age, obesity, diabetes mellitus, cigarette smoking, and low education levels – are associated with higher odds of clinical attachment loss. Integrating oral health care into CVD control programs is essential. Patients with known CVD risk factors should receive plaque control, oral prophylaxis, and regular caries risk assessments. Preventive measures such as fissure sealants and resin restorations can help halt the progression of early carious lesions.
Prince *et al* ^[^34^]^	South Africa	128	Mean age of 44.24 (14.55) and 49.98 (10.45)	Case-control cohort	16S rDNA gene sequencing using subgingival plaque samples	*Campylobacter gracilis* was significantly associated.	Disruption of the oral microbiota is associated with metabolic syndrome and systemic inflammation. This disruption may act as a gateway to systemic diseases such as diabetes and cardiovascular diseases. Significant changes include an increased abundance of gram-positive aerobic and anaerobic microorganisms.
Ojehanon and Akhionbare^[^35^]^	Nigeria	1200	18–77 years	Cross-sectional study	Sphygmomanometer and stethoscope	19.7%	Some dental patients are unaware of their blood pressure levels. Screening all dental patients for hypertension is crucial to prevent potential complications.
Blankson *et al* ^[^36^]^	Ghana	175	18–86 years	Cross-sectional study	Screening	31.4%	Dental clinics play a critical role in identifying undiagnosed hypertension and diabetes. Clinics can also detect individuals with inadequate control of previously diagnosed blood pressure and blood sugar levels. With effective referral and follow-up systems, dental offices can serve as valuable hubs for hypertension and diabetes screening.
Amougou *et al* ^[^37^]^	Cameroon	154	≥21 years	Cross-sectional study	Periodontal status was observed in subjects aged 21 and over with cardiovascular disease. Cardiovascular diagnosis was made by a cardiologist, following the recommendations of the European Society of Cardiology.	59.7%	This study shows that half of the population had a weakened periodontal state, attributed to poor individual oral hygiene and lack of professional assistance. Several authors recognize an increased risk of cardiovascular disease in patients with periodontal disease, independent of other cardiovascular risk factors. A multidisciplinary approach, including the oral health component, is crucial for improving the management of patients with cardiovascular disease.
Yamori *et al* ^[^38^]^	Tanzania	81	46–58 years	Cross-sectional study	Blood pressure (BP) and anthropometric measurements 24-hour urine collection Blood sampling Dental examination Lifestyle questionnaires	Not reported	Low dietary potassium intake, often coupled with low fiber intake, increases blood pressure (BP) and periodontal inflammation. Periodontitis may elevate BP through chronic inflammation and oxidative stress mechanisms. Maintaining oral health and traditional dietary habits, including higher potassium intake from fruits and vegetables, can reduce the risks of hypertension (HTN) and periodontitis.
Arowojolu *et al* ^[^39^]^	Nigeria	100	20–88 years	Cross-sectional study	Cardiovascular risk factors were compared with periodontal parameters	70%	A significant relationship exists between blood pressure and mean carotid artery intima-media thickness, relative to oral hygiene status. Regular preventive dental visits should be emphasized to reduce cardiovascular health risks.
Umeizudike *et al*^[^40^]^	Nigeria	1910	18–88 years	Retrospective, cross-sectional survey	Patients’ hospital records included age, sex, BP, and history of hypertension	39.9%	Hypertension is highly prevalent among routine dental patients. Routine blood pressure assessments should be conducted before dental procedures.
Amougou *et al* ^[^41^]^	Cameroon	80	≥21 years	Cross-sectional comparative study	Participants: Hypertensive (test group) Non-hypertensive (comparison group) Periodontal indices measured during examination: Plaque index Bleeding index Loss of attachment	80 participants (40 hypertensives and 40 controls) were enrolled.	This study suggests a link between periodontal disease and essential hypertension.
Umeizudike *et al* ^[^42^]^	Nigeria	150	23–77 years	Comparative, cross-sectional study	Periodontal clinical parameters Body mass index (BMI) 75 hypertensive individuals 75 gender-matched normotensive controls	150 participants (75 hypertensives and 75 gender-matched normotensive controls)	Moderate gingivitis is a predictor of hypertension, alongside known hypertension risk factors. Hypertensive individuals exhibit a greater extent of periodontal pockets compared to non-hypertensives. This study underscores the link between preventable, low-grade periodontal inflammation and hypertension.
Eno Belinga *et al* ^[^43^]^	Cameroon	558	30–85 years	Prospective observational study	Medical history and oral examination	28.9% of patients had CVD, Hypertension accounted for 87.6% of CVD, and it was concerning 141 (25.3%) patients in the sample	Periodontal diseases are linked to cardiovascular diseases. Enhancing oral health can be incorporated into cardiovascular disease control programs. Oral health improvement could also support broader efforts to combat chronic non-communicable diseases, the leading cause of global mortality.

### Synthesis of results

This scoping review highlights the potential association between dysbiotic oral microbiomes and HTN in SSA^[^32–43^]^. Most studies included in the review indicate that imbalances in the oral microbiome, often associated with conditions like periodontal diseases and dental caries, may contribute to the development or exacerbation of HTN. These studies covered a range of participant groups, including dental patients without a known history of HTN, individuals unaware of their HTN status, and those already diagnosed with HTN. The reported prevalence of HTN among these groups varied widely, from 19.7 to 91.8%, demonstrating the significant burden of HTN in SSA and its possible link with oral health^[^32–43^]^.

Toward dental caries experience, it was found that people with poorly controlled systolic and DBP had higher odds of experiencing caries^[^33^]^. In addition, an alteration in the periodontal status of subjects with CVDs was reported^[^37^]^. Where reported, the mean gingival index in hypertensive subjects ranged between 1.02 ± 0.38 and 1.5 ± 0.485, the mean plaque index was between 1.3 ± 0.48 and 1.9 ± 0.590, and the mean clinical attachment loss ranged between 0.49 ± 0.56 and 2.8 ± 1.859^[^32,37,41,42^]^. The periodontal conditions reportedly associated with HTN or CVDs in the included studies were gingivitis^[^35,42,43^]^, acute periapical periodontitis^[^35^]^, periodontal disease in general^[^41^]^, and periodontitis^[^32,38,39,43^]^.

### Quantitative associations between oral conditions and HTN

Of the 12 included studies, nine reported quantitative effect estimates for the association between oral conditions (periodontitis, gingivitis, dental caries, or specific oral bacteria) and HTN or blood pressure parameters (Table 2).

**Table 2:** 

Author/year	Country	Sample size	Study design	Reported prevalence of hypertension or species identified	Key quantitative finding (OR, RR, or correlation)	95% CI	*P*-value
Leye *et al* ^[^32^]^	Senegal	246	Case-control study	246 patients (123 hypertensive patients and 123 non-hypertensive patients)	OR: 2.045	[1.188–2.128]	0.001
Oyapero *et al* ^[^33^]^	Nigeria	268	Cross-sectional study	91.8% of HTN with poor DBP and SBP	OR: 0.73 for DBP for dental caries OR: 0.72 for SBP for dental caries OR: 0.93 for DBP for clinical attachment loss OR: 0.85 for SBP for dental caries	[0.61,0.86] [0.59,0.90] [0.84,1.06] [0.74,1.04]	0.017 0.045 0.175 0.683
Prince *et al* ^[^34^]^	South Africa	128	Case-control cohort	*Campylobacter gracilis* was significantly associated	OR: 0.29	[0.12, 0.68]	0.005
Ojehanon and Akhionbare ^[^35^]^	Nigeria	1200	Cross-sectional study	19.7%	Not reported	Not reported	Not reported
Blankson *et al* ^[^36^]^	Ghana	175	Cross-sectional study	31.4%	Not reported	Not reported	Not reported
Amougou *et al* ^[^37^]^	Cameroon	154	Cross-sectional study	59.7%	Not reported	Not reported	Not reported
Yamori *et al* ^[^38^]^	Tanzania	81	Cross-sectional study	Not reported	Regression coefficient (β) on SBP (0.288) to periodontitis Regression coefficient (β) for DBP (0.293) to periodontitis	Not reported	0.018 0.015
Arowojolu *et al* ^[^39^]^	Nigeria	100	Cross-sectional study	70%	Pearson correlation of SBP (0.389) to simplified oral hygiene index Pearson correlation on DBP (0.256) to simplified oral hygiene index Pearson correlation on SBP (0.312) to Gingival index Pearson correlation on DBP (0.247) to Gingival index	Not reported	0.000 0.010 0.002 0.013
Umeizudike *et al* 2013^[^40^]^	Nigeria	1910	Retrospective, cross-sectional survey	39.9%	Not reported	Not reported	Not reported
Amougou *et al* ^[^41^]^	Cameroon	80	Cross-sectional comparative study	80 participants (40 hypertensives and 40 controls) were enrolled	OR: 1.5 for plaque index OR: 1.11 for bleeding index OR: 1.5 for attachment loss	[1.3–1.97] [1.1–2.3] [1.2–3.1]	0.042 0.001 <0.001
Umeizudike *et al* ^[^42^]^	Nigeria	150	Comparative, cross-sectional study	150 participants (75 hypertensives and 75 gender-matched normotensive controls)	OR: 3.314 for moderate gingivitis	[1.156–9.502]	0.026
Eno Belinga *et al* ^[^43^]^	Cameroon	558	Prospective observational study	28.9% of patients had CVD, hypertension accounted for 87.6% of CVD, and it concerned 141 (25.3%) patients in the sample	OR: 4.30 for gingivitis OR: 2.87 for periodontitis	[1.85–10.00] [1.04–7.93]	0.001 0.04

Among case-control studies, Leye *et al* ^[^32^]^ reported a significant odds ratio (OR) of 2.045 (95% CI: 1.188–2.128; *P* = 0.001) for the association between periodontal disease and HTN in Senegal. Prince *et al* ^[^34^]^ identified *Campylobacter gracilis* as significantly associated with metabolic syndrome and cardiovascular risk in South Africa (OR: 0.29; 95% CI: 0.12–0.68; *P* = 0.005). In a prospective study, Eno Belinga *et al* ^[^43^]^ reported significant associations in Cameroon for gingivitis (OR: 4.30; 95% CI: 1.85–10.00; *P* = 0.001) and periodontitis (OR: 2.87; 95% CI: 1.04–7.93; *P* = 0.04) with HTN.

Among cross-sectional studies, Oyapero *et al* ^[^33^]^ found that dental caries was significantly associated with reduced odds of both DBP (OR: 0.73; 95% CI: 0.61–0.86; *P* = 0.017) and SBP (OR: 0.72; 95% CI: 0.59–0.90; *P* = 0.045) in Nigeria. Amougou *et al* ^[^41^]^ reported significant odds ratios for plaque index (OR: 1.5; 95% CI: 1.3–1.97; *P* = 0.042), bleeding index (OR: 1.11; 95% CI: 1.1–2.3; *P* = 0.001), and attachment loss (OR: 1.5; 95% CI: 1.2–3.1; *P* < 0.001) among hypertensive patients in Cameroon. Umeizudike *et al* ^[^42^]^ found that moderate gingivitis was significantly associated with HTN in Nigeria (OR: 3.314; 95% CI: 1.156–9.502; *P* = 0.026). Yamori *et al* ^[^38^]^ reported significant positive regression coefficients between periodontitis and both systolic (*β* = 0.288; *P* = 0.018) and diastolic (*β* = 0.293; *P* = 0.015) blood pressure. Arowojolu *et al* ^[^39^]^ demonstrated significant positive correlations between oral hygiene indices and blood pressure measurements (*P* values ranging from 0.000 to 0.013).

Four cross-sectional studies^[^35–37,40^]^ reported the prevalence of HTN among their study populations (19.7, 31.4, 39.9, and 59.7%, respectively), but did not provide effect estimates for the association between oral conditions and HTN.

## Discussion

### Interpretation of findings

This scoping review examines the current literature on the role of oral microbial dysbiosis in the development of HTN. Globally, there is notable evidence for this association and a lack of evidence in some regions, particularly in SSA.

Research suggests that dysbiosis in the oral microbiome may influence immune responses and contribute to oral diseases, especially periodontal diseases^[^21,44^]^. The studies included in this review comprise dental patients without a history of HTN, individuals unaware of their HTN status, and those already diagnosed with HTN^[^32–43^]^. Additionally, these studies assessed the oral health of participants, some of whom had been diagnosed with oral diseases such as periodontal disease and dental caries. Periodontitis is a complex inflammatory disease that primarily affects the supporting structures of the teeth^[^45^]^. It can lead to damage to blood vessel endothelial cells and result in endothelial dysfunction^[^46^]^. Elevated levels of C-reactive protein (CRP), interleukin-6 (IL-6), and neutrophils in the bloodstream have been linked to periodontitis^[^47^]^. Recent research has connected several inflammatory markers to the onset of HTN, including CRP, IL-6, and tumor necrosis factor-alpha (TNF-α)^[^48,49^]^.

The systemic response to periodontal infection could be a reasonable explanation for the reported association between periodontitis and an increased risk of HTN. Furthermore, the risk of cardiac or cerebrovascular events may increase due to elevated inflammatory factors, which may also increase inflammatory activity in lesions associated with atherosclerosis^[^50^]^. Patients with atherosclerosis and periodontitis have been found to harbor some of these microbes in their arteries and are at high risk for CVD^[^51,52^]^. Moreover, this association has reportedly been found in older and postmenopausal women^[^53,54^]^. It is important to note that all included studies in this review were observational in design. Consequently, the following discussion describes associations only, and no causal relationships should be inferred. With this limitation in mind, most studies have reported an association between a dysbiotic oral microbiome and HTN, either as an isolated condition or as part of broader CVDs^[^32–43^]^. For instance, using 16S ribosomal Deoxyribonucleic Acid (rDNA) gene sequencing, Prince *et al* demonstrated that, in metabolic syndrome, the oral microbiome was disrupted and contributed to systemic diseases, including CVDs, among individuals in SSA^[^34^]^.

The reported prevalence of HTN ranged from 19.7 to 91.8%, underscoring the significant health concern HTN poses in SSA in relation to oral health status^[^32–43^]^. As described in the literature, the increasing rates of HTN in SSA have highlighted the need for prevention and health promotion programs related to CVDs and associated risk factors. In addition, research has revealed a substantial correlation between HTN and a dysbiotic oral microbiome, even in the absence of other causative factors^[^55,56^]^. Research has explored and strongly supported the association between HTN and oral diseases^[^57^]^. This correlation emphasizes the need for better education and knowledge regarding periodontal treatment and preventive care for people who are at risk of HTN^[^58^]^. In this review, several studies emphasize the importance of improving oral health to reduce HTN and suggest that dental clinics can play a crucial role in detecting undiagnosed CVD and risk factors^[^35,36,40,43^]^. These studies recommend integrating oral health care into CVD control programs and encouraging regular preventive dental visits to mitigate cardiovascular risk^[^32,33^]^. Additionally, they advocate for routine blood pressure assessments for hypertensive patients prior to dental procedures^[^40^]^. Given the general lack of awareness of HTN among dental patients, it is recommended that all dental patients be screened.

Cross-sectional, case-control, cohort, and prospective observational designs were the primary designs employed in the studies. Although these designs are beneficial for identifying associations, they do not establish a causal relationship between oral microbial dysbiosis and HTN. Furthermore, numerous studies failed to account for confounding factors, including age, gender, socioeconomic status, and lifestyle behaviors, which could potentially impact both oral health and HTN outcomes. The reliability of the associations observed may be compromised by this lack of rigorous control. The capacity to draw definitive conclusions about the causal role of dysbiotic oral microbiomes in HTN is further restricted by the absence of longitudinal studies with repeated measures or randomized controlled trials (RCTs).

Geographical limitations were also noted, with relevant studies being conducted in a limited number of SSA countries, such as South Africa, Nigeria, Tanzania, Senegal, Ghana, and Cameroon, often in urban settings^[^32–43^]^. This limitation affects the generalizability of the findings across the diverse populations and environments of SSA. Additionally, differences in the diagnostic criteria for oral diseases and HTN across studies posed challenges in directly comparing results.

Diagnostic heterogeneity was also substantial. HTN definitions varied, with some studies using 140/90 mmHg and others not specifying thresholds. Periodontal assessment ranged from full-mouth clinical attachment loss to simplified indices, and no study employed standardized case definitions. These differences preclude direct comparison and meta-analytic synthesis.

### Quantitative synthesis of findings

The quantitative data extracted from 8 of the 12 included studies revealed several notable patterns. First, effect sizes varied considerably across studies, with odds ratios ranging from 0.29 (an association with *C. gracilis*) to 4.30 (increased risk for gingivitis)^[^34,43^]^. This wide range likely reflects differences in study designs, outcome definitions, and population characteristics rather than true heterogeneity in biological effects. Second, the strongest and most consistent associations were observed for periodontitis and gingivitis. Eno Belinga *et al*^[^43^]^ reported the largest effect size (OR: 4.30 for gingivitis), followed by Umeizudike *et al*^[^42^]^ (OR: 3.314 for moderate gingivitis) and Leye *et al*^[^32^]^ (OR: 2.045 for periodontal disease). These findings support the hypothesized role of periodontal inflammation in systemic vascular dysfunction.

Third, the associations reported by Oyapero *et al*^[^33^]^ between dental caries and blood pressure (ORs <1.0) are counterintuitive and warrant cautious interpretation. The authors suggested that dental caries may be associated with dietary factors (e.g., sugar intake) that confound the relationship with blood pressure, rather than representing a direct protective effect of caries itself. Fourth, the absence of effect estimates in four cross-sectional studies^[^33–37,40^]^ limits the ability to quantify the strength of association in those populations. This gap highlights the need for future studies to report standardized effect measures (e.g., odds ratios with confidence intervals) to enable meta-analytic synthesis. Finally, the confidence intervals for several estimates were wide (e.g., Umeizudike *et al*^[^42^]^, 95% CI: 1.156–9.502), reflecting small sample sizes in some studies and limiting precision. Larger, well-designed studies are needed to produce more stable estimates of the association between oral dysbiosis and HTN in SSA populations.

### Research gaps and implications for future research

Despite the growing recognition of the relationship between oral health and systemic diseases, including HTN, the existing research in SSA has primarily concentrated on associations between specific oral diseases–notably periodontal disease and HTN. One study provided species-level data. Prince *et al*^[^34^]^ identified *C. gracilis* as significantly associated with metabolic syndrome and cardiovascular risk in a South African population (OR: 0.29; 95% CI: 0.12–0.68; *P* = 0.005). This finding is notable because *C. gracilis* is an anaerobic, gram-negative rod previously implicated in periodontal disease and systemic inflammation^[^59,60^]^. However, the direction of this association (OR < 1.0) is unexpected and warrants further investigation.

Studies conducted outside SSA have identified specific oral microbes, including *Porphyromonas gingivalis, Aggregatibacter actinomycetemcomitans, Treponema denticola*, and *Fusobacterium* species, as significant contributors to the pathogenesis of HTN and CVD^[^19,28,44,61^]^. These bacteria have been detected in atherosclerotic plaques, with strong correlations to elevated blood pressure and cardiovascular pathology^[^21,27,62^]^. In contrast, research from SSA lacks detailed analysis of the oral microbiome’s composition and its functional role in the onset of HTN, primarily focusing on general associations with periodontal diseases. This absence of species-level data from SSA populations represents a critical knowledge gap. This gap can be addressed through advanced molecular studies employing 16S rRNA gene sequencing or metagenomic approaches to characterize the oral microbiome composition and identify region-specific microbial signatures associated with HTN. This could provide critical insights into the oral microbiome’s role in HTN within African populations.

This gap presents a significant opportunity for more studies to be conducted in SSA. To establish a stronger link between oral microbiome dysbiosis and high blood pressure, we could focus on certain types of microbes and how they cause disease. Learning about these processes in the local population could also show how different areas have different microbial patterns influenced by genetic, environmental, and dietary factors. These differences may play a role in the development of high blood pressure in SSA. This kind of study would help us learn more about how oral health affects cardiovascular health in SSA. It would also show that monitoring the oral microbiome should be part of programs that aim to prevent CVDs.

### Limitations

In this scoping review, the literature search was restricted to papers accessible online, published in English, and from SSA. This restriction may lead to selection bias by excluding relevant scientific studies published in other languages. Additionally, the heterogeneity of study designs, diagnostic criteria, and population characteristics poses a challenge to the generalizability of the results and limits the reliability of pooled conclusions. Therefore, no meta-analysis was performed, and the wide variation in HTN prevalence (19.7–91.8%) likely reflects methodological inconsistency rather than true population differences. The scarcity of longitudinal cohort studies and RCTs on the role of a dysbiotic oral microbiome in the development of HTN in SSA emphasizes the need for more comprehensive research in this area. The absence of a formal risk of bias assessment means the results should be interpreted as hypothesis-generating rather than causal, as confounding factors (e.g., lifestyle, socioeconomic status) could not be systematically ruled out. In addition, studies employing observational designs are inherently susceptible to residual confounding and reverse causality, which prevents causal inference from being drawn from the observed associations between oral microbiome dysbiosis and HTN. Future research should overcome the limitations noted in the majority of studies conducted in this particular area and worldwide in order to draw more accurate conclusions regarding the role of the dysbiotic oral microbiota in the development of HTN in SSA. These drawbacks include the absence of untreated control groups, brief follow-up times, small sample sizes, and confounding variables.

In addition, the generalizability of the findings is limited by the geographic and demographic scope of the included studies. Relevant studies were conducted in only six of the 46 SSA countries: South Africa, Nigeria, Tanzania, Senegal, Ghana, and Cameroon. No studies were identified from East African countries, Central African countries, or Lusophone African countries. Furthermore, most studies recruited participants from urban or peri-urban settings, with little to no representation of rural populations, who comprise a substantial proportion of SSA residents and face different healthcare access, dietary patterns, and environmental exposures. Therefore, the findings of this review cannot be generalized to the entire SSA region and should be considered exploratory and hypothesis-generating for understudied subregions.

## Conclusion

This scoping review presents preliminary, hypothesis-generating evidence suggesting an association between a dysbiotic oral microbiome and HTN in SSA. The most available evidence from SSA supports an association between periodontal disease and HTN. However, direct evidence on the oral microbiome itself remains very limited, with only one study performing species-level microbial analysis. In addition, the predominance of cross-sectional studies precludes causal conclusions. The growing number of people with HTN in this group, including those who are not aware they have it, underscores the urgency of this problem. In order to prevent and control HTN in SSA, additional research is required to clarify the mechanisms underlying this link and to develop targeted interventions through oral health initiatives.

## Data Availability

Data sharing is not applicable to this article as no datasets were generated during the current study.

## Research registration unique identifying number (UIN)

This scoping review was registered with the Open Science Framework (https://osf.io/w6nvg).
